# Identifying Boys With Autism Spectrum Disorder Based on Whole-Brain Resting-State Interregional Functional Connections Using a Boruta-Based Support Vector Machine Approach

**DOI:** 10.3389/fninf.2022.761942

**Published:** 2022-02-22

**Authors:** Lei Zhao, Yun-Kai Sun, Shao-Wei Xue, Hong Luo, Xiao-Dong Lu, Lan-Hua Zhang

**Affiliations:** ^1^Centre for Cognition and Brain Disorders, The Affiliated Hospital of Hangzhou Normal University, Hangzhou, China; ^2^Institute of Psychological Science, Hangzhou Normal University, Hangzhou, China; ^3^Zhejiang Key Laboratory for Research in Assessment of Cognitive Impairments, Hangzhou, China; ^4^Department of Psychiatry, Sir Run Run Shaw Hospital, Zhejiang University School of Medicine, Hangzhou, China; ^5^College of Medical Information and Engineering, Shandong First Medical University and Shandong Academy of Medical Sciences, Tai'an, China

**Keywords:** autism spectrum disorder, support vector machine, resting-state functional magnetic resonance neuroimaging (R-fMRI), functional connection (FC), Boruta

## Abstract

An increasing number of resting-state functional magnetic resonance neuroimaging (R-fMRI) studies have used functional connections as discriminative features for machine learning to identify patients with brain diseases. However, it remains unclear which functional connections could serve as highly discriminative features to realize the classification of autism spectrum disorder (ASD). The aim of this study was to find ASD-related functional connectivity patterns and examine whether these patterns had the potential to provide neuroimaging-based information to clinically assist with the diagnosis of ASD by means of machine learning. We investigated the whole-brain interregional functional connections derived from R-fMRI. Data were acquired from 48 boys with ASD and 50 typically developing age-matched controls at NYU Langone Medical Center from the publicly available Autism Brain Imaging Data Exchange I (ABIDE I) dataset; the ASD-related functional connections identified by the Boruta algorithm were used as the features of support vector machine (SVM) to distinguish patients with ASD from typically developing controls (TDC); a permutation test was performed to assess the classification performance. Approximately, 92.9% of participants were correctly classified by a combined SVM and leave-one-out cross-validation (LOOCV) approach, wherein 95.8% of patients with ASD were correctly identified. The default mode network (DMN) exhibited a relatively high network degree and discriminative power. Eight important brain regions showed a high discriminative power, including the posterior cingulate cortex (PCC) and the ventrolateral prefrontal cortex (vlPFC). Significant correlations were found between the classification scores of several functional connections and ASD symptoms (*p* < 0.05). This study highlights the important role of DMN in ASD identification. Interregional functional connections might provide useful information for the clinical diagnosis of ASD.

## Introduction

Autism spectrum disorder (ASD) is a neurodevelopmental disability characterized by persistent deficits in social communication associated with restricted and repetitive patterns of behavior, interests, or activities (Minshew and Williams, 2007). Individuals with ASD have difficulty with everyday reciprocal social communication and social interactions, causing heavy burdens to individuals, families, and society (Van Loo and Martens, 2007). However, the neural correlates underlying ASD symptoms have not been fully elucidated, and further examination is warranted to gain a more comprehensive understanding of this disorder. Resting-state functional MRI (R-fMRI), which measures the blood oxygen level-dependent (BOLD) signal recorded at rest, has emerged as a promising tool for exploring disorder-related brain function alterations (Biswal, 2012; Lau et al., 2019). Previous R-fMRI studies have demonstrated dispersively distributed functional disruptions in ASD, while this disorder has been increasingly characterized as the dysregulation of brain networks with altered functional connectivity (Hull et al., 2017). Connectivity is a general description of the interactive relationship between two independent brain regions in anatomical space. Functional connectivity aims to describe this relationship from the point of functional synchronization. In the scan of R-fMRI, functional connectivity is measured by statistical dependence in BOLD fluctuations between two brain regions or brain networks. Except for anatomical morphology, the human brain regions can also be subdivided by functional characteristics. In a previous study (Dosenbach et al., 2010), the 160 regions of interest (ROIs) covering the whole brain were labeled and grouped into six networks, namely, the default mode network (DMN), frontoparietal network (FPN), cingulo-opercular network (CON), sensorimotor network (SMN), occipital network (ON), and cerebellum network (CN). This brain atlas was defined based on activation patterns of the brain across different cognitive tasks and may support additional information for interpreting developmental changes in brain function.

According to a previous study, there was a decrease in the functional connectivity of the medial prefrontal cortex (mPFC) with left anterior insula in patients with ASD (Von dem Hagen et al., 2013). In contrast to healthy subjects, patients with ASD exhibited decreased posterior cingulate cortex (PCC) connectivity with the superior frontal gyrus and increased connectivity of which with the parahippocampal gyrus (Monk et al., 2009). Moreover, associations between functional connectivity and ASD symptoms have been observed. For instance, the connectivity between the left anterior insula and bilateral precuneus showed a negative correlation with autism symptom severity (Xu et al., 2018). Atypical functional interactions between the core regions of the DMN, including the PCC and the mPFC, were correlated with impaired social function in patients with ASD (Lynch et al., 2013; Von dem Hagen et al., 2013). These findings indicated that the clinical characteristics of ASD might be represented in aberrant functional interactions in large-scale brain networks.

Machine learning has attracted an increasing amount of attention in R-fMRI studies as a promising technique for identifying patients with neuropsychiatric diseases at the individual level (Geng et al., 2018; Riaz et al., 2018). Previous studies have suggested that functional interactions in large-scale brain networks could be utilized for the classification of patients vs. healthy subjects. For example, interregional functional connections were regarded as discriminative features of support vector machine (SVM) to differentiate patients diagnosed with attention deficit hyperactivity disorder (ADHD) from healthy subjects (Sun et al., 2020). Whole-brain functional connections were also adopted as features of SVM to identify patients with schizophrenia (Li et al., 2019). Machine learning has been regarded as an exploratory framework to characterize the brain functional organization from interregional functional connections that may be implicated in neuropathology underlying neuropsychiatric disorders. For example, the cerebellum showed high discriminative power for identifying patients with ADHD (Sun et al., 2020). The functional connections across the DMN and visual cortical areas showed high discriminative power when discriminating major depressive patients from healthy subjects (Zeng et al., 2012). Despite progress in patient identification using machine learning, it is not yet clear how functional interactions in large-scale brain networks serve as highly discriminative features to realize the classification of ASD.

“Boruta” algorithm (Kursa et al., 2010) preserves the feature set with statistically significant contributions to classification rather than highest contributions and, thus, reduces the overfitting. In this study, the Boruta algorithm was used to determine the ASD-related functional connectivity pattern. Based on this pattern, we distinguished patients with ASD from typically developing controls (TDC) using SVM and then characterized brain network and regions with high discriminative power. Considering that the DMN has been shown to be significantly involved in ASD (Padmanabhan et al., 2017), we expected that the DMN would play an important role in ASD identification.

## Materials and Methods

### Participants

Table 1 shows the characteristics of all participants in this study. A total of 48 boys with ASD and 50 typically developing age-matched male controls were included. These participants were part of the New York University (NYU) Langone Medical Center dataset on the Autism Brain Imaging Data Exchange (ABIDE) platform (http://fcon_1000.projects.nitrc.org/indi/abide/). The exclusion criteria were as follows: (1) female sex; (2) not between 7 and 18 years old; and (3) excessive head motions. Considering that gender imbalance might interfere with the ASD analysis, we regarded sex as a potential confounding factor and employed a relatively careful participant selection criterion by choosing only male subjects.

**Table 1:** Demographic and clinical data.

**Groups**	**ASD (mean ± SD)**	**TDC (mean ± SD)**	***p*-value**
No. of subjects	48	50	
Sex (M/F)	48/0	50/0	
Age	11.02 ± 2.65	11.96 ± 2.87	0.090
FIQ	107.92 ± 16.66	111.92 ± 14.30	0.200
VIQ	104.81 ± 14.96	112.16 ± 12.94	0.011^*^
PIQ	110.19 ± 18.79	108.90 ± 15.85	0.710
ADI S	19.85 ± 5.10		
ADI C-V	15.34 ± 3.84		
ADOS T	11.71 ± 4.28		
ADOS C	3.56 ± 1.64		
ADOS S	8.15 ± 2.97		

*FIQ, full IQ; PIQ, performance IQ; VIQ, verbal IQ; ADI S, ADI-R social total; ADI C-V, ADI-R communication total for verbal children; ADOS T, ADOS total; ADOS C, ADOS communication total; ADOS S, ADOS social total*.

* *indicates p < 0.05 after two-sample t-test*.

The Diagnostic and Statistical Manual of Mental Disorders, Fourth Edition (DSM-IV-TR) was used to diagnose ASD. This study was carried out in accordance with the principles of the Declaration of Helsinki and was approved by the Institutional Review Board (IRB) of NYU and the NYU School of Medicine. Prior to participation, informed consent was obtained from all participants and their parents/legal guardians (for participants < 18 years).

### Image Preprocessing

All images were acquired using a Siemens MAGNETOM Allegra syngo 3.0 T MR Scanner (Siemens AG, Medical Solutions, Erlangen, Germany). A 8:07 min T1-weighted sagittal MP-RAGE structural image was obtained (flip angle = 7°, time of repetition (TR) = 2,530 ms, time of echo (TE) = 3.25 ms, number of volumes = 256, voxel size = 1.3 × 1 × 1.3 mm, FOV = 256 mm, slice thickness = 1.33 mm, and T1 = 1,100 ms). A 6-min R-fMRI scan was obtained using a T2^*^-weighted gradient-echo EPI pulse sequence (flip angle = 90°, number of slices = 33, TR = 2,000 ms, TE = 15 ms, number of volumes = 180, voxel size = 3 × 3 × 4 mm, FOV = 240 mm, and slice thickness = 4 mm). During the R-fMRI scan, participants were asked to relax with their eyes open, while a white cross-hair against a black background was projected on a screen.

Data processing was performed using a combination of DPABI (http://www.rfmri.org/), SPM (http://www.fil.ion.ucl.ac.uk/spm/), and custom code written in MATLAB. For each subject, the first 10 volumes of functional images were discarded to allow for magnetization equilibration effects and the adaptation of the participants to the scan circumstances. The remaining images were corrected for slice timing and motion. All participants in this study had a maximum displacement of < 2 mm in the *x*-, *y*-, or *z*-axes and an angular motion of <2°. The corrected images were then normalized into a standard stereotactic space as defined by the Montreal Neurological Institute (resampling voxel size = 3 × 3 × 3 mm) and smoothed using a 6-mm full-width at half-maximum Gaussian kernel. To further reduce the effects of confounding factors, we also regressed out the nuisance signals (Friston-24 motion parameters, white matter signal, and cerebrospinal fluid signal). Finally, functional images underwent temporal band-pass filtering ([0.01–0.08 Hz]).

### Boruta Feature Selection

The Boruta algorithm was adopted based on the interregional functional connections for feature selection before ASD identifying in this study. Interregional functional connections were calculated based on the functional brain atlas (Dosenbach et al., 2010). First, the average R-fMRI time series of 160 ROIs of the atlas (Dosenbach et al., 2010) were extracted to calculate functional connections between all possible pairs of ROIs. Specifically, the functional connections between two ROIs were obtained by calculating the Pearson's correlation coefficient between their average R-fMRI time series. Then, correlation coefficients were subsequently converted to *z*-values by Fisher's *r*-to-*z* transformation to improve the normality of values. Finally, a series of 160 × 160 symmetric matrices represented the whole-brain network of participants, and the upper triangle elements of the matrix were extracted for feature selection.

As a feature selection method built based on a random forest classifier, the Boruta method selects the features that have significantly more relevance with classification than randomly permuted features (Kursa et al., 2010). Compared with general feature selection methods that focus on the so-called “minimal-optimal” problem by obtaining the best possible classification results with possibly minimal feature sets, the Boruta method aims to identify all features that are relevant for classification. In this study, we employed the Boruta method to examine the ASD-related functional connectivity pattern (implemented in Python: https://github.com/scikit-learn-contrib/boruta_py). In brief, the method can be described as follows: each feature of the original feature matrix A is shuffled across subjects to generate shadow features to add randomness and remove correlations between features and class labels, and then, the shadow feature matrix B is concatenated with the original feature matrix A to form a mixture feature matrix C (C = [A, B]) in which the number of features is twice that of the original feature matrix A. A random forest classifier is performed on the mixture feature matrix C (the Gini value was utilized as the cost function; the number of the decision tree was set as 500 in this study). The Gini importance of each feature is calculated. Real features with Gini importance higher than the 95th percentile of shadow features will be assigned a hit and then a statistical test (using a binomial distribution with *p* = 0.05, two-step correction: FDR and Bonferroni correction) will be performed to mark features as “confirmed” or “rejected.” The features marked as “rejected” will be removed from original feature matrix A. The above steps are repeated until each feature has a mark or the number of iterations reaches the predefined criterion (the maximum of iterations was set as 250 in this study).

We applied a *k*-means clustering method based on functional connections to classify the aforementioned ASD-related connections into several patterns. Specifically, a matrix with the ASD-related connections as rows and the subjects as columns was constructed. The rows of this matrix were then classified into different clusters based on their distributions across subjects. The connections assigned into the same cluster constitute a unique pattern of connection that covary across subjects.

The brain functional connection network has amounts of nodes and functional interactions and can be represented as a graph. The topological complexity of a brain functional connection network that reflects the global performance of a network was generally characterized with graph theory measures (Bullmore and Sporns, 2012). ASD-related functional connectivity pattern was a sparse presentation of the whole brain and also a specific representation for the classification of ASD. In this study, we aimed to employ the network degree (ND) to describe the topological characteristics of this pattern. The ND of a brain network was defined as the number of direct connections between a predefined brain network and other networks, representing the relative importance of a brain network in regard to the information flow in the brain (Zuo et al., 2012).

### SVM Classification

**Figure 1:**
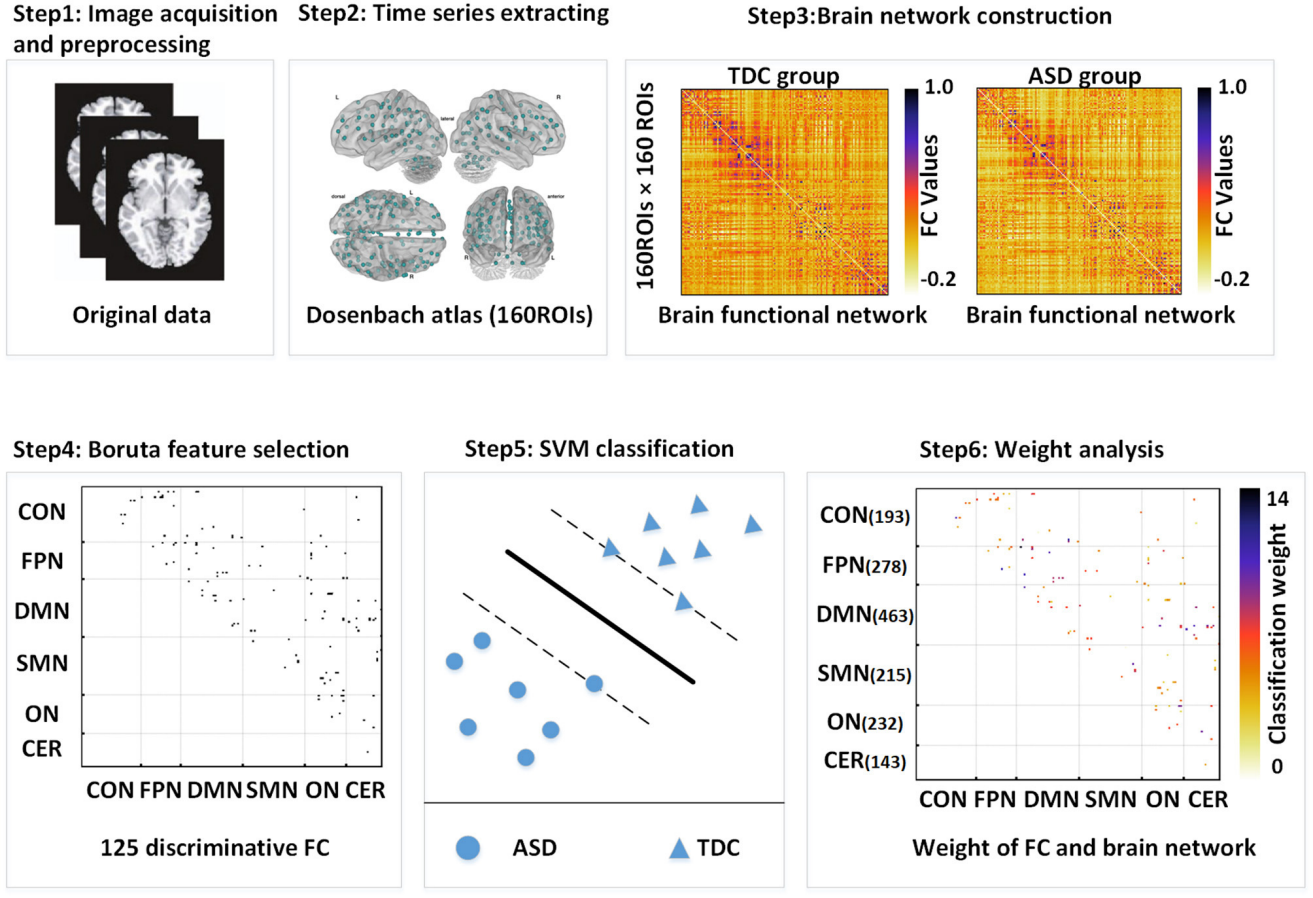
The Boruta-SVM process flowchart. FC, functional connection; SVM, support vector machine; DMN, default mode network; FPN, frontoparietal network; CON, cingulo-opercular network; SMN, sensorimotor network; ON, occipital network; CN, cerebellum network.

Figure 1 shows a flowchart for SVM classification including the construction of whole-brain interregional functional connections, Boruta feature selection, and SVM classification. We defined the ASD-related functional connections after the Boruta feature selection as classification features and used SVM with the radial basis function (RBF) kernel function to distinguish patients with ASD from TDC (implemented using “libsvm”: https://www.csie.ntu.edu.tw/~cjlin/libsvm/). SVM is the most common machine learning technique for the clinical application study of R-fMRI. When the cost of sample acquisition is expensive, SVM displays a key advantage due to its algorithm principle. The hyperplane of SVM used to distinguish different categories can be determined by fewer support vectors and thus exhibit excellent performance in small samples usually. The classification weight of a functional connection was obtained by averaging weights across all trials of leave-one-out cross-validation (LOOCV). We defined the classification weight of a brain region as the sum of the classification weight of all the connections to and from that brain region. The classification weight of each brain network was evaluated by summing the classification weight of all the regions within that brain network. The brain regions whose classification weights were two SDs higher than the average classification weight of brain regions contributing to classification were defined as the important brain regions.

### Statistical Analysis and Permutation Tests

The Pearson correlation analyses were performed between the classification scores of the functional connections and ASD symptoms including the Autism Diagnostic Observation Schedule (ADOS: ADOS_total, ADOS_communication, and ADOS_social) and Autism Diagnostic Interview-Revised (ADI_R: ADI_R_social total and ADI_R_verbal total). The *p* = 0.05 was set as the statistical threshold. We employed a permutation test to evaluate the statistical significance of classification accuracy (Golland and Fischl, 2003). Specifically, after randomly permuting the labels corresponding to the training samples, we trained the classifier on the permuted training samples and then performed validation. The permutation process was conducted 10,000 times in total. It is assumed that classification performance is reliable when the generalization rate obtained by the classifier trained on the real class labels is higher than the 95% CI of the classifier trained on randomly relabeled class labels. To comprehensively evaluate the classification performance, we also assessed sensitivity and specificity. These two measures are commonly utilized together to estimate the predictive performance of a classification model. The sensitivity indicates the proportion of positive predictions in the group of patients, and the specificity indicates the proportion of negative predictions in the group of healthy controls.

## Results

### Classification Results

**Figure 2:**
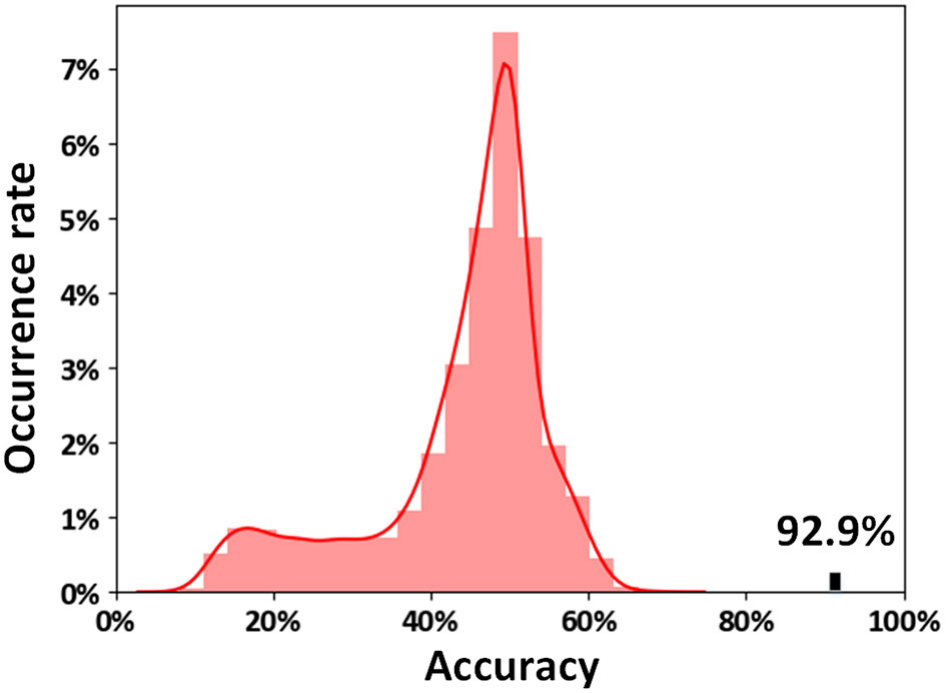
Permutation distribution of the classification accuracy estimate. The permutation was repeated 10,000 times, resulting in 10,000 classification accuracies based on random labels. The classification accuracy (92.9%) based on the true labels exceeded each of the classification accuracies resulting from the permutation, indicating that our classifier can reliably learn the relationship between the features and the labels with a probability higher than the predetermined 95% criterion.

The results indicated that 92.9% of subjects were correctly classified using an SVM method by LOOCV (sensitivities = 95.8% and specificities = 90.0%). The classification features were extracted using the Boruta method, and a total of 125 ASD-related functional connections were selected. The distribution of the permutation tests (10,000 times) showed that our classification accuracy (92.9%) was significantly higher than the random level (*p* < 0.0001, Figure 2).

### Brain Regions and Networks With High Classification Weights

**Figure 3:**
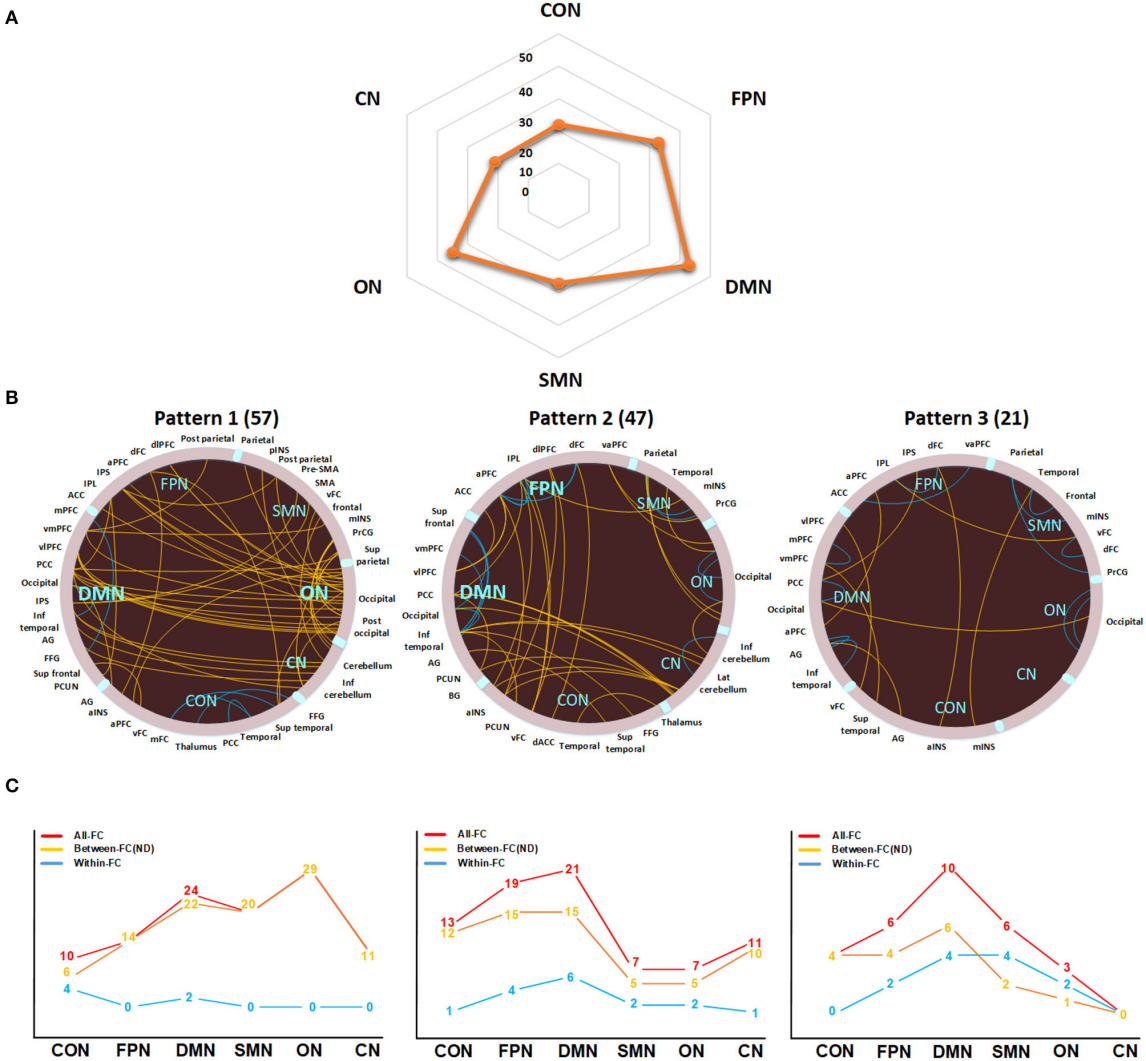
Network degree (ND) analysis of ASD-related functional connections. **(A)** The ND distribution of six networks. **(B)** The three functional connectivity patterns identified by *k*-means clustering. Patterns 1, 2, and 3 consist of 57, 47, and 21 functional connections, respectively. The yellow and blue lines in circles indicate between-network and within-network connections, respectively. The yellow lines in the line charts indicate the ND or the number of between-network connections for each network. The red and blue lines in the line charts indicate the number of functional connections and within-network functional connections for each network, respectively. FC, functional connection; DMN, default mode network; FPN, frontoparietal network; CON, cingulo-opercular network; SMN, sensorimotor network; ON, occipital network; CN, cerebellum network; a, anterior; d, dorsal; dl, dorsolateral; inf, inferior; Lat, lateral; med, medial; post, posterior; sup, superior; v, ventral; vm, ventromedial; vl, ventrolateral; ACC, anterior cingulate cortex; AG, angular gyrus; BG, basal ganglia; FFG, fusiform gyrus; INS, insula; IPL, inferior parietal lobe; IPS, intraparietal sulcus; mINS, middle insula; PCC, posterior cingulate cortex; PCUN, precuneus; PFC, prefrontal cortex; PrCG, precentral gyrus; SMA, supplementary motor area.

The DMN exhibited the highest ND, followed by the FPN and ON (Figure 3A). As shown in Figure 3B, three interregional connectivity patterns were detected by *k*-means clustering based on the ASD-related functional connections. Specifically, the DMN and ON showed relatively higher ND in pattern 1, while relatively higher ND was found in the DMN and FPN in pattern 2. Pattern 3 showed lower ND in each network and a higher proportion of within-network connections than other patterns.

**Figure 4:**
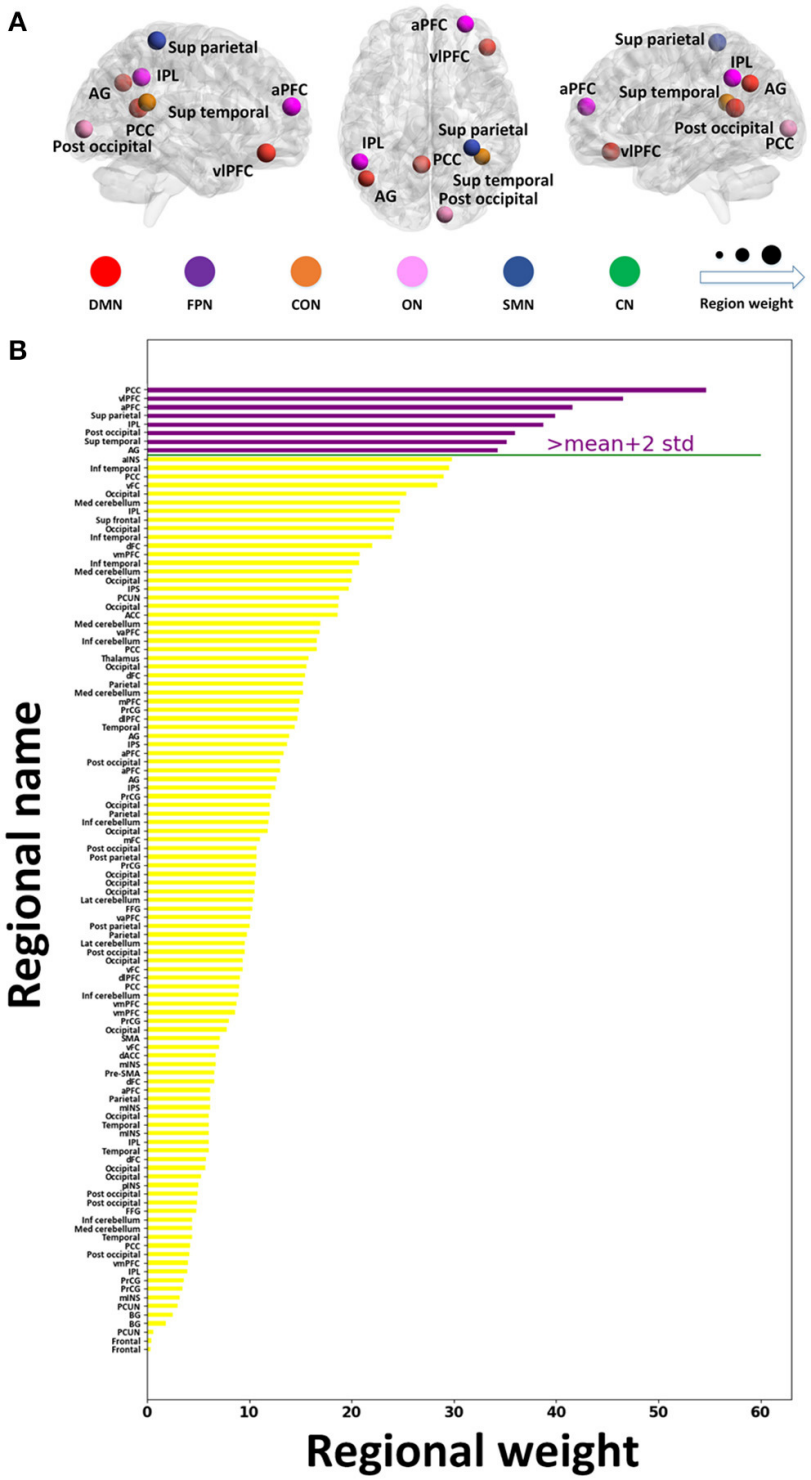
Regional importance in the resting-state network. **(A)** The location of the top eight important brain regions. **(B)** Regional importance ranking. The important regions whose classification weights are two SDs above the mean classification weights of all the discriminative regions are presented with purple bars. The y- and x-labels represent the regional name and classification weight, respectively. a, anterior; d, dorsal; dl, dorsolateral; inf, inferior; Lat, lateral; med, medial; post, posterior; sup, superior; v, ventral; vm, ventromedial; vl, ventrolateral; ACC, anterior cingulate cortex; AG, angular gyrus; BG, basal ganglia; FFG, fusiform gyrus; INS, insula; IPL, inferior parietal lobe; IPS, intraparietal sulcus; mINS, middle insula; PCC, posterior cingulate cortex; PCUN, precuneus; PFC, prefrontal cortex; PrCG, precentral gyrus; SMA, supplementary motor area.

As shown in Figure 4, eight important brain regions were identified and ranked by classification weights, including the PCC, ventrolateral prefrontal cortex (vlPFC), anterior prefrontal cortex, superior parietal lobe, inferior parietal lobe, posterior occipital lobe, superior temporal sulcus, and angular gyrus. Most regions were part of the DMN or FPN. Furthermore, the DMN and FPN exhibited higher classification weights.

**Figure 5:**
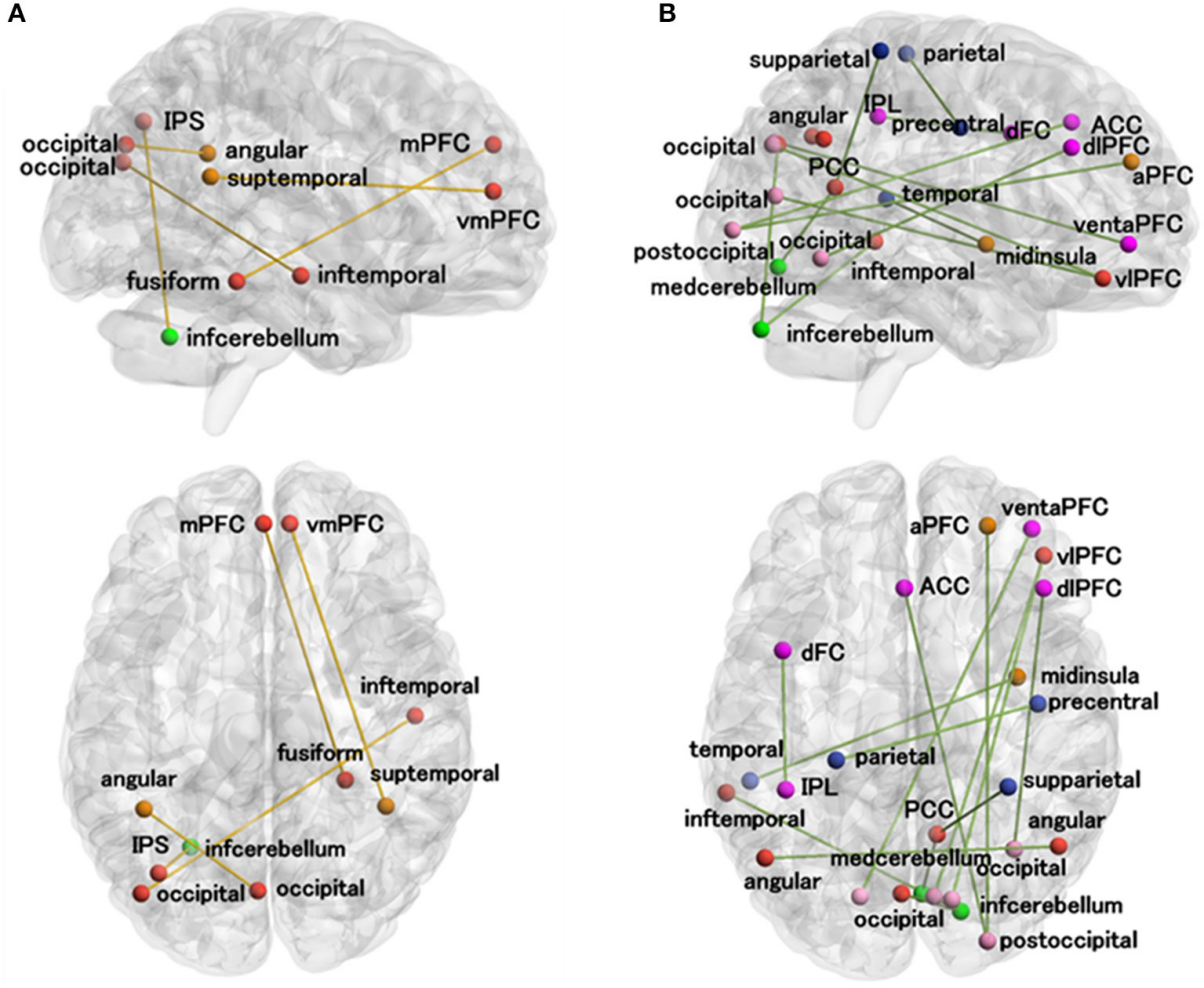
Correlations between the functional connection classification scores and clinical scales in patients with ASD. **(A)** The functional connections whose classification scores were negatively correlated with at least one of the ASD measures (ADI_R_social total, ADI_R_verbal total, or ADOS_communication). **(B)** The functional connections whose classification scores were positively correlated with at least one of the ASD measures (ADI_R_social total, ADI_R_verbal total, ADOS_total, ADOS_social total, and ADOS_communication). The meanings of the regional colors are the same as in Figure 3B. a, anterior; d, dorsal; inf, inferior; post, posterior; sup, superior; dl, dorsolateral; vm, ventromedial; v, ventral; vl, ventrolateral; ACC, anterior cingulate cortex; AG, angular gyrus; fusiform gyrus, FFG; IPL, inferior parietal lobe; IPS, intraparietal sulcus; ITG, inferior temporal gyrus; med, medial; mINS, middle insula; mPFC, medial prefrontal cortex; PCC, posterior cingulate cortex; PFC, prefrontal cortex; PrCG, precentral gyrus.

As shown in Figure 5; Table 2, there was a significantly negative correlation (*p* < 0.05) between the classification scores of five DMN-related functional connections and ASD symptoms measured by clinical scales (ADI_R_social total, ADI_R_verbal total, or ADOS_communication) in patients with ASD. The classification scores of fourteen functional connections were positively correlated (*p* < 0.05) with ASD symptoms (ADI_R_social total, ADI_R_verbal total, ADOS_total, ADOS_social, or ADOS_communication) in patients with ASD.

**Table 2:** Correlations between classification score and clinical scales of functional connections in ASD.

**ROI-ROI**	**Network**	**Scale**	***r*-value**	***p*-value**
**Positive correlations**				
Post occipital—aPFC	ON—CON	ADI_R_V	0.369	0.011
Occipital—vaPFC	ON—FPN	ADI_R_V	0.323	0.027
Occipital—vlPFC	ON—DMN	ADI_R_V	0.416	0.004
Occipital—vlPFC	ON—DMN	ADI_R_V	0.324	0.026
Post occipital—ACC	ON—FPN	ADI_R_V	0.357	0.014
Occipital—dlPFC	ON—FPN	ADI_R_S ADI_R_V	0.350 0.300	0.017 0.041
IPL—dFC	FPN—FPN	ADOS_C	0.300	0.043
Temporal—mINS	SMN—CON	ADOS_C	0.383	0.009
Parietal—PrCG	SMN—SMN	ADOS_S	0.318	0.028
AG—AG	DMN—DMN	ADOS_T ADOS_C	0.296 0.299	0.041 0.044
PCC—Sup parietal	DMN—SMN	ADI_R_S ADOS_T ADOS_C ADOS_S	0.302 0.336 0.300 0.301	0.042 0.020 0.043 0.038
ITG—Inf cerebellum	CN—DMN	ADOS_T ADOS_C ADOS_S	0.458 0.389 0.419	0.001 0.008 0.003
PCC—Med cerebellum	CN—DMN	ADOS_C	0.314	0.034
Occipital—Inf cerebellum	CN—DMN	ADI_R_V	0.356	0.015
**Negative correlations**				
FFG—mPFC	DMN—DMN	ADI_R_S	−0.330	0.025
vmPFC—Sup temporal	DMN—CON	ADOS_C	−0.321	0.030
Occipital—Inf temporal	DMN—DMN	ADI_R_S	−0.312	0.035
Occipital—AG	DMN—CON	ADI_R_V	−0.353	0.015
IPS—Inf cerebellum	DMN—CN	ADI_R_V	−0.311	0.033

*Positive correlations: functional connections whose classification score was positively correlated with at least one of the ADI_R_social total, ADI_R_verbal total, ADOS_total, ADOS_social, and ADOS_communication; negative correlations: functional connections whose classification score was negatively correlated with at least one of the ADI_R_social total, ADI_R_verbal total, and ADOS_communication*.

*DMN, default mode network; FPN, frontoparietal network; CON, cingulo-opercular network; SMN, sensorimotor network; ON, occipital network; CN, cerebellum network; a, anterior; d, dorsal; inf, inferior; med, medial; post, posterior; sup, superior; v, ventral; vm, ventromedial; vl, ventrolateral; ACC, anterior cingulate cortex; AG, angular gyrus; FFG, fusiform gyrus; IPL, inferior parietal lobe; IPS, intraparietal sulcus; ITG, inferior temporal gyrus; mINS, middle insula; mPFC, medial prefrontal cortex; PCC, posterior cingulate cortex; PFC, prefrontal cortex; PrCG, precentral gyrus; ADI_R_S, ADI_R social total; ADI_R_V, ADI_R_verbal total; ADOS_T, ADOS_total; ADOS_C, ADOS_communication; and ADOS_S, ADOS_social*.

## Discussion

In this study, we distinguished patients with ASD from TDC based on resting-state interregional functional connections using a Boruta-SVM approach. A high classification accuracy of 92.9% was achieved based on the classification features of ASD-related functional connections by means of the Boruta feature selection method. The DMN exhibited a high ND with a larger classification weight. The eight important brain regions with high classification weights, including the PCC, vlPFC, aPFC, superior parietal lobe, inferior parietal lobe, posterior occipital lobe, superior temporal sulcus, and angular gyrus, were primarily located in the DMN and FPN. Thereinto, the PCC and vlPFC presented relatively high discriminative power. We found statistically significant correlations between the classification scores of several ASD-related functional connections and ASD symptoms. The DMN was negatively correlated with ASD symptoms. These findings highlighted an important role of the DMN in distinguishing ASD patients from TDC.

We proposed the Boruta method to select features reflecting ASD-related functional connectivity patterns. The Boruta method recognizes features relevant to the target and was used to construct an interpretable predictive model (Kursa et al., 2010). Most of the previous combined SVM and R-fMRI studies were mainly dedicated to identifying features that were useful for making accurate predictions, but the limitation of those studies was that the interpretability of the predictive model was seldom assessed (Wee et al., 2012). We employed the ND to describe classification features selected using the Boruta method. The highest ND was found in the DMN and might suggest a predominance in the ASD-related functional connectivity pattern. According to the results of the weight analysis, we found that these functional connections involved in the DMN were of high discriminative power during SVM classification, highlighting the importance of the DMN. These findings were consistent with previous results showing an abnormal DMN connectivity pattern in ASD (Cheng et al., 2015; Abbott et al., 2016). For example, patients with ASD exhibited reduced network integration (reduced within-network connections) and increased out-of-network connections for the DMN (Abbott et al., 2016). Furthermore, increased functional connectivity between the DMN and salience network was associated with a higher cognitive impairment in ASD (Abbott et al., 2016). Together, these findings suggest that the DMN might be considered a relevant locus for ASD identification.

The FPN exhibited a relatively higher classification weight and ND in pattern 2, indicating a potential high contribution to the ASD-related functional connectivity pattern. The FPN is crucial for coordinating behavior in an accurate, rapid, and flexible goal-driven manner (Bareham et al., 2018; Fiebelkorn et al., 2018). A growing number of studies implicate the FPN as a neural substrate for impaired executive function, which is a frequently reported symptom of ASD, thus prompting researchers to further examine the potential contribution of the FPN to the underlying pathophysiology of ASD. For example, a significantly decreased FPN-insular participation coefficient was found in children with ASD during a task with executive function demand (Lynch et al., 2017). A previous study demonstrated that patients with ASD exhibited lower levels of network integration in the FPN and that reduced FPN connectivity was related to attention deficit symptoms in the ASD group (Solomon et al., 2009), indicating that functional connection disruptions of the FPN involved in the clinical symptoms of ASD.

This study found that the PCC and vlPFC were the two most discriminative regions. As a central region of the DMN, The PCC showed abnormalities in many neurological and psychiatric disorders including ASD (Sun et al., 2012; Yang et al., 2016). For example, ASD exhibited hypoconnectivity between the PCC and other regions within the DMN (Weng et al., 2010). Moreover, the hyperconnectivity of the PCC with the parahippocampal gyrus has been shown to relate with the severity of ASD symptoms measured by the ADOS (Lynch et al., 2013). The vlPFC is an important region in the FPN that involves goal-appropriate response selection and response inhibition (Aron et al., 2004). Patients with ASD showed significantly decreased brain activation in the vlPFC when participating in a temporal discounting task (Murphy et al., 2017). The functional connection of the vlPFC with the anterior cingulate cortex was associated with the performance of a cognitive control task (Solomon et al., 2014). Compared with TDC, patients with ASD had weaker activation in the occipital area which is mostly associated with face processing (Renzi et al., 2015). Increasing evidence suggests associations between abnormal face processing and impaired social function in ASD (Nomi and Uddin, 2015). A study combining DTI and fMRI found that abnormal structural connectivity and functional activation of the occipital cortex were related to social communication deficits in patients with ASD (Jung et al., 2019).

In the ASD group, several classification scores of functional connections were significantly correlated with ASD symptoms, indicating that the ASD-related functional connectivity pattern has the potential to reflect symptom severity. Notably, the functional connections whose classification scores were correlated with ASD symptoms were widely distributed throughout the whole brain, further supporting the hypothesis that the clinical characteristics of ASD were represented in aberrant functional interactions in large-scale brain networks (He et al., 2007; Fiebelkorn et al., 2018). Functional connections whose classification scores were negatively correlated with ASD symptoms were connected from/to the DMN, which might imply a close relationship between the DMN and ASD symptoms.

Previous studies proposed different pipelines to classify ASD from TDC, while most efforts were made to improve accuracy, and the clinical relevance was partly overlooked. This study further decodes the connectivity patterns which could be used to distinguish ASD from TDC. In addition, we not only replicated the important role of the DMN in ASD from the classification of the disease but also identified the relationships between autistic symptom severity and classification weight of the DMN. These results suggested that the clinical relevance of features served more focus in the machine learning study of R-fMRI studies.

Notably, the functional connections whose classification scores were correlated with ASD symptoms were widely distributed throughout the whole brain, further supporting the hypothesis that the clinical characteristics of ASD were represented in aberrant functional interactions in large-scale brain networks (He et al., 2007; Fiebelkorn et al., 2018). Functional connections whose classification scores were negatively correlated with ASD symptoms were connected from/to the DMN, which might imply a close relationship between the DMN and ASD symptoms.

Two limitations should be considered. First, only male patients were included in the present analysis because ASD is more prevalent among males than among females in the general population; however, this might limit the generalizability of our findings. Therefore, further studies should take into account the relationship between gender and ASD. Second, this study demonstrates that the DMN might play a key role in ASD; however, how the DMN specifically interacts with ASD symptoms needs more in-depth research.

## Conclusion

We demonstrated that the ASD-related functional connections identified by the Boruta method could be regarded as features to differentiate patients with ASD from TDC. This finding indicated that resting-state interregional functional connections might provide neuroimaging-based information to clinically assist with the diagnosis of ASD. In particular, the DMN and its core region (the PCC) exhibited high discriminative power for identifying ASD, thereby highlighting the important role of the DMN in understanding the potential pathophysiology of ASD.

## Data Availability Statement

Publicly available datasets were analyzed in this study. This data can be found here: Autism Brain Imaging Data Exchange (ABIDE), http://fcon_1000.projects.nitrc.org/indi/abide/.

## Ethics Statement

The studies involving human participants were reviewed and approved by Hangzhou Normal University. Written informed consent to participate in this study was provided by the participants' legal guardian/next of kin.

## Author Contributions

LZ and Y-KS analyzed the data and summarized the results. LZ and S-WX organized the manuscript and wrote the first draft of the manuscript. X-DL and L-HZ revised the manuscript. All authors have read and approved the final manuscript.

## Funding

This study was supported by the Zhejiang Medical and Health Science and Technology Project (2022KY1055), the Natural Science Foundation of Zhejiang Province (LY17H180007), the Key Medical Disciplines of Hangzhou, and The Affiliated Hospital of Hangzhou Normal University.

## Conflict of Interest

The authors declare that the research was conducted in the absence of any commercial or financial relationships that could be construed as a potential conflict of interest.

## Publisher's Note

All claims expressed in this article are solely those of the authors and do not necessarily represent those of their affiliated organizations, or those of the publisher, the editors and the reviewers. Any product that may be evaluated in this article, or claim that may be made by its manufacturer, is not guaranteed or endorsed by the publisher.
